# Prevalence of hepatitis B and clinical outcomes in inflammatory bowel disease patients in a viral-endemic region

**DOI:** 10.1186/s12876-016-0516-2

**Published:** 2016-08-22

**Authors:** Heyson C. H. Chan, Vincent W. S. Wong, Grace L. H. Wong, Whitney Tang, Justin C. Y. Wu, Siew C. Ng

**Affiliations:** Department of Medicine and Therapeutics, Institute of Digestive Disease, Li Ka Shing Institute of Health Science, State Key Laboratory of Digestive Diseases, Chinese University of Hong Kong, Hong Kong, Hong Kong SAR, China

**Keywords:** Inflammatory bowel disease, Hepatitis B, Immunosuppression

## Abstract

**Background:**

Little is known of the prevalence of hepatitis B virus (HBV) infection and its effect on choice of therapy and disease course in patients with inflammatory bowel disease (IBD). We assessed the prevalence of HBV in Hong Kong as well as determinants of altered transaminases, effects of HBV infection on therapeutic strategy and clinical course in IBD.

**Methods:**

In this retrospective cohort, hepatitis B surface antigen (HBsAg), liver function tests, and IBD disease characteristics were recorded. Logistic regression was used to identify factors associated with altered transaminases.

**Results:**

Four hundred six IBD patients were recruited. HBV infection was found in 5.7 % patients in Hong Kong. The use of steroids (OR, 2.52; *p* = 0.010) and a previous history of surgery (OR 2.33; *p* = 0.026) were associated with altered transaminases in IBD. There was no significant difference in disease control and use of IBD medication between HBsAg-positive and HBsAg-negative IBD patients.

**Conclusion:**

The prevalence of HBV among patients with IBD in Hong Kong (5.7 %) is similar to that of general population (~7 %). There was no difference in disease control and use of IBD medication between subjects with or without HBV.

## Background

Previously a disease predominantly of the West, there is now a rising incidence and prevalence of inflammatory bowel disease (IBD) in Asia [1, 2]. Immunosuppressive therapy is the mainstay of therapy of IBD. However, it can be associated with complications such as the reactivation of hepatitis B virus (HBV) [3–5]. This is of particular importance in Asian countries which have a moderate to high prevalence of HBV infection [6]. Several studies from the West have reported the prevalence of HBV infection in IBD patients [7–10]. However current data on whether IBD patients have a higher risk of HBV infection have been conflicting. There is also a paucity of data on the prevalence of HBV infection among IBD patients in Southeast Asia.

International guidelines recommend that all hepatitis B surface antigen (HBsAg)-positive IBD patients should receive anti-viral prophylaxis before starting immunosuppressive agents [11–14]. However, the risk of reactivation appeared to be related to the type and magnitude of immunosuppression [15]. The American Gastroenterological Association Institute (AGA) recently recommends that only patients at moderate to high risk undergoing immunosuppressive therapy should have anti-viral prophylaxis [16]. However, there is still a paucity of data in supporting this new recommendation.

In addition, it is currently unclear if the presence of HBV infection in IBD patients influence the disease behavior or clinical course of IBD itself. It has been reported that IBD patients with chronic HBV have a worse prognosis than their non-infected counterparts due to the infrequent use of immunosuppressant [17].

In this study we assessed the prevalence of HBV infection in patients with IBD in ethnically Chinese individuals from Hong Kong. We also evaluated the determinants of altered transaminases and the effect of HBV infection on the therapeutic strategy and clinical course of IBD patients.

## Methods

### Patients

In this retrospective cohort study, all IBD patients aged 18 years or older with a diagnosis of Crohn’s disease (CD) or ulcerative colitis (UC) for at least 3 months defined by histology, endoscopy or radiology attending the IBD clinic at the Prince of Wales Hospital from the period of June 2012 to June 2013 were included. All patients had their hepatitis B status checked during the study period.

They were further assessed to investigate the determinants of altered transaminases in IBD patients, the characteristics of hepatitis B patients with altered transaminases and to compare IBD patients with and without hepatitis B.

Patients were followed up at 3- to 6-monthly intervals. The clinical phenotypes of IBD were classified according to the Montreal Classification [18] and disease activity was recorded prospectively at each visit. Assessment was based on the physician’s global assessment, taking into account the patient’s symptoms, inflammatory markers and recent endoscopic assessment. Disease control was recorded as well-controlled or not well-controlled. Electronic hospital record was reviewed for a history of IBD related surgery and hospital admissions. A history of IBD-associated liver disease (e.g., primary sclerosing cholangitis) was recorded. Active smoker was defined as subjects who smoke at least 1 cigarette daily in the past 6 months. Ex-smoker was defined as patients who smoked at least 1 cigarette daily but had quitted for at least 6 months. Non-smoker was defined as patients who had never smoked. All patients were tested negative for hepatitis A and hepatitis C.

Blood tests including complete blood count, renal and liver function tests, inflammatory markers [Erythrocyte Sedimentation Ratio (ESR) or C reactive protein (CRP)] were monitored during each clinic visit. The use of IBD medications including 5-aminosaliylic acid (5ASA), corticosteroids, thiopurine (azathioprine/6-mercaptopurine), methotrexate and anti-tumor necrosis factor antibody (Infliximab or Adalimumab) were reviewed from the time of diagnosis until last follow-up. The duration and dosage of these medications were recorded.

Chronic hepatitis B was defined as positive HBsAg for more than 6 months. Patients who had a positive HBsAg were tested for hepatitis e antigen (HBeAg) and serum HBV-DNA. We did not include patients with occult hepatitis B (anti-HBc positive; HBsAg negative) patients as the risk of hepatitis flare in occult HBV patients was rare in patients receiving common medications for IBD. If the patient had been put on antiviral agents, their baseline HBV-DNA level was recorded. A history of liver cirrhosis and hepatocellular carcinoma (HCC) were recorded. A diagnosis of liver cirrhosis was made by clinical, laboratory and imaging criteria while that of HCC was confirmed by tumor markers, imaging +/− biopsy results.

All IBD patients with HBV were invited to have their liver stiffness measured by transient elastography. The use of anti-viral agents for the treatment of chronic hepatitis B, including the dosage and duration were recorded.

Abnormal liver function (altered transaminases) was defined as serum Alanine Aminotransferase (ALT) level twice the upper limit of normal (ULN) (i.e., ALT >110 IU/mL).

In order to distinguish between altered transaminases due to IBD and reactivation of hepatitis B, in IBD patients with hepatitis B who had altered transaminases, details of the episode of liver enzymes elevation were reviewed. Medications used at the time of altered transaminases and the HBV DNA levels were recorded. Altered transaminases related to HBV reactivation was considered probable when an increase in ALT was observed in the absence of other potential causative factors (including other viral hepatitis) and HBV DNA values were elevated.

#### Transient elastography

Fibroscan (Echosens, Paris, France) was performed by the principal investigator (HC) who had received formal accredited training. Fibroscan were performed at the time of study. The median of 10 successful acquisitions was kept as representative of liver stiffness. Liver stiffness measurements were considered reliable only if 10 successful acquisitions were obtained and the interquartile range to median ratio of the 10 acquisitions was < 30 %. Advanced liver fibrosis and cirrhosis were defined according to the transient elastography algorithm for chronic hepatitis B previously validated against liver histology. Patients with normal ALT and liver stiffness >9.0 kPa or raised ALT (1-5x ULN) and liver stiffness >12.0kPa were considered to have liver fibrosis and those with normal ALT and liver stiffness >12.0 kPa or raised ALT (1-5x ULN) and liver stiffness >13.4 kPa were considered to have liver cirrhosis [19].

#### Matching with chronic hepatitis B patients without IBD

Cases (HBsAg-positive IBD) were defined as IBD patients tested positive for HBsAg. Controls consisted of HBV patients without IBD, selected from a cohort of previous hepatitis B studies. Controls were matched 3:1 to cases by age (+/− 3 years), gender, HBeAg status and use antiviral agents [20]. As the major risk factors for HBV infection in Asia are through vertical transmission, patients usually acquire the infection early in childhood. With matching of age, the duration of HBV infection is likely similar between cases and controls. Liver stiffness and HBV DNA level (baseline HBV DNA level if patient had been put on anti-viral agents) were recorded. Differences in liver stiffness and HBV DNA level between cases and controls were assessed.

### Statistical analysis

Results were expressed as mean with standard deviation (SD) or number- with percentages. Unpaired *t*-test was used to test the differences in continuous variables and Chi-squared test for categorical variables. Univariate analysis was used to identify factors associated with altered transaminases. Factors with a *P* value of less than 0.1 were included in the multivariate analysis, performed by binary logistic regression. Risks were expressed as odds ratio (OR) with 95 % confidence interval (CI). A *p* value <0.05 was considered significant. All statistical analyses were performed using SPSS 14.0 for Windows software package.

## Results

### Demographics and characteristics of IBD

Four hundred six IBD patients were recruited (185 CD and 221 UC). The characteristics of the IBD patients in Hong Kong are tabulated in Table 1. The median time of follow up from diagnosis was 8 years (range 1–29)

**Table 1:** Clinical demographics of IBD patients in the Hong Kong cohort

Variables	Crohn’s disease (*n* = 185)	Ulcerative colitis (*n* = 221)
Age (mean ± SD)	40.6 ± 14.3	48.4 ± 14.0
Age at diagnosis (mean ± SD)	32.1 ± 14.1	38.6 ± 13.1
Sex [male, n (%)]	125 (67.6)	116 (52.5)
Smoking history		
Active smoker Ex-smoker Non-smoker	16 (8.6) 30 (16.2) 139 (75.1)	17 (7.7) 29 (13.1) 175 (79.2)
Montreal classification (n, %)		
Disease location		
Ileum Colon Ileocolon	34 (18.3) 47 (25.4) 104 (56.3)	--
Upper GI involvement	38 (20.5)	--
Disease behavior		
Inflammatory Stricturing^a^ Penetrating^a^	82 (44.3) 53 (28.6) 67 (36.2)	--
Perianal involvement	32 (17.2)	--
Disease extent		
Proctitis Left sided UC Pancolitis	--	67 (30.3) 65 (29.4) 89 (40.3)

*IBD*, inflammatory bowel disease, *CD* Crohn’s disease, *UC* ulcerative colitis

^a^Not mutually exclusive

One patient had IBD-associated liver disease (namely, primary sclerosing cholangitis) in our cohort. This patient had cholestatic liver dysfunction with alkaline phosphatase fluctuating between 150 and 300 IU/L. She did not experience any episodes of altered transaminases.

### Prevalence of HBsAg positivity

In Hong Kong, 5.7 % IBD patients had positive HBsAg (6.5 % CD; 5.0 % in UC).

### Determinants of altered transaminases

Altered transaminases were observed in 58 IBD patients (14.4 %). Among the patients with history of altered transaminases, the median ALT was 199 IU/L (range 112 to 2520). None of the patients had liver failure or required liver transplantation. Results of univariate analysis are shown in Table 2a. In multivariate analysis, the use of steroid [OR 2.524, 95 % confidence interval (CI) 1.398–6.450] and a previous history of IBD related surgery (OR 2.330, 95 % CI 1.107–4.906) were independently associated with of altered transaminases (Table 2b).

**Table 2:** Univariate and multivariate analysis of determinants of altered transaminases

a) Univariate analysis				
Variables	No liver dysfunction (*n* = 348)	Liver dysfunction (*n* = 58)	OR	*P* value
Age, years (Mean ± SD)	44.6 ± 15.1	46.6 ± 12.1	--	0.333
Age at diagnosis, years (Mean ± SD)	35.5 ± 14.0	36.8 ± 13.7	--	0.481
Sex, male (n; %)	206 (59.2 %)	35 (60.3)	0.953	0.869
Crohn’s vs ulcerative colitis (ratio)	0.775	1.43	0.588	0.063
Previous surgery (n; %)	42 (12.1)	16 (27.6)	2.776	0.002
Use of 5-ASA (n; %)	177 (50.9)	17 (29.3)	0.401	0.003
Use of thiopurines (n; %)	130 (37.3)	31 (53.4)	1.908	0.024
Use of steroids (n; %)	42 (12.1)	18 (31.0)	3.279	<0.001
Use of anti-TNF agents (n; %)	7 (2.0)	2 (3.4)	1.74	0.497
HBsAg positivity (n; %)	17 (4.9)	6 (10.3)	2.24	0.104
HBV and thiopurines (n; %)	10 (3)	3 (5.2)	1.833	0.369
HBV and steroids (n; %)	3 (0.6)	3 (1.7)	3.035	0.368
HBV and anti-TNF agents (n; %)	1 (0.3)	1 (1.7)	6.088	0.204
b) Multivariate analysis				
	OR (95 % CI)	*P* value		
Crohn’s vs ulcerative colitis	0.834 (0.467–1.858)	0.589		
Previous Surgery	2.330 (1.107–4.906)	0.026		
5-ASA	0.428 (0.197–1.163)	0.058		
Steroids	2.524 (1.394–6.450)	0.010		
Thiopurines	0.698 (0.307–1.695)	0.411		

*5ASA* 5-aminosalicylic acid

### Clinical outcomes of HBsAg-positive and HBsAg-negative IBD Patients

There was no significant difference between HBV and non-HBV IBD patients in terms of disease location and behavior (Table 3). There was a significantly higher rate of use of biologics in patients with positive HBsAg (*p* = 0.030), but there was no significant difference in the use of other IBD medications. One HBsAg-negative patient died during the study period because of chest infection.

**Table 3:** Comparison of clinical demographics between IBD patients according HBsAg positivity

	HBsAg positive (*n* = 23)	HBsAg negative (*n* = 383)	*P* value
Age (Mean ± SD)^a^	44.1 ± 9.5	44.9 ± 13.9	0.783
Sex (Male) (n; %)^b^	12 (52.2)	228 (59.5)	0.775
Crohn’s vs ulcerative colitis (ratio)^b^	1.09	0.823	0.512
Previous surgery (n; %)^b^	5 (21.7)	53 (13.8)	0.298
Disease Flare (n; %)^b^	8 (8.7)	59 (10.6)	0.770
Admission (n; %)^b^	7 (30.4)	69 (18)	0.145
5ASA (n; %)^b^	8 (34.8)	186 (48.5)	0.199
Thiopurine (n; %)^b^	13 (56.5)	148 (38.6)	0.093
Steroid (n; %)^b^	6 (26.1)	54 (14.1)	0.116
Biologics (n; %)^b^	2 (8.7)	7 (1.8)	0.030

*5ASA* 5-aminosalicylic acid

^a^unpaired *t*-test

^b^Chi Square test

### IBD patients with HBV

Among the 23 IBD patients with HBV, 12 (52.2 %) of them were men, with a mean age of 44.1 years (SD, 9.5). The mean age at the time of IBD diagnosis was 31 years (SD, 13.9). The median time of follow up among IBD patients with HBV was 13 years (range 3–24 years). Of the 23 HBsAg-positive IBD patients, 3 (13.0 %) were HBeAg positive. Six of the patients were receiving antiviral agents, one was receiving lamivudine and five were receiving entecavir. None of our patients received interferon treatment. 18 (78.3 %) patients had detectable HBV DNA and 10 (23.4 %) of them had HBV DNA >2000 IU/mL. The mean serum HBV DNA level was 3.41log IU/mL (SD, 2.10) in HBsAg positive IBD patients which is similar to HBsAg positive controls without IBD (4.12 log IU/mL; SD, 1.69; *p* = 0.319). 1 (4.3 %) HBsAg positive IBD patients had cirrhosis and 6 (8.7 %) HBsAg positive controls without IBD had cirrhosis (*p* = 0.494). The rate of HCC were also comparable between HBsAg positive IBD patients (0; 0 %) and HBsAg positive controls without IBD (1; 1.5 %) (*p* = 0.494).

The 13 patients who had immunosuppressive therapy, 5 (38.4 %) were also receiving antiviral prophylaxis. There was no difference in the mean HBV DNA level in patients who were receiving immunosuppressants compared with those who were not [3.89 log IU/mL (SD, 2.23) versus 3.57log IU/mL (SD, 2.14); *p* = 0.731].

### Cause of altered transaminases in HBsAg-positive IBD patients

The characteristics of IBD patients with HBV who experienced altered transaminases were summarized in Table 4*.* None of these patients had evidence of IBD-associated liver disease.

**Table 4:** Characteristics of HBsAg positive IBD patients with altered transaminases

Age	Sex	IBD	HBeAg	Highest ALT	Bilirubin at time of highest ALT	Albumin at time of highest ALT	HBV DNA level at time of altered transaminase	IBD medications	Possible cause of altered transaminase	Use of antiviral prophylaxis at time of altered transaminase
33	M	CD	-ve	176	17	40	4.21 log IU/mL	5ASA	Likely immune clearance phase with hepatitis B e-seroconversion	No
38	M	CD	+ve	146	14	38	7.85log IU/mL	Thiopurines, steroid	Possible hepatitis B reactivation vs azathioprine related liver dysfunction	No
53	M	UC	+ve	2520	55	32	7.07log IU/mL	5ASA, Steroid	Possible hepatitis B reactivation	No
46	M	CD	-ve	162	12	38	4.02log IU/mL	Thiopurines	Transient liver dysfunction with uncertain cause	No
32	F	UC	-ve	207	9	41	2.44log IU/mL	5ASA	Likely related to hyperemesis gravidum	No
36	F	CD	+ve	592	11	26	Undetectable	Thiopurines, Steroid, Biologics,	Possibly related to TPN; unlikely related to hepatitis B reactivation	Yes

*IBD* inflammatory bowel disease, *CD* Crohn’s disease, *UC* ulcerative colitis, *ALT* Alanine Aminotransferase, *HBV* hepatitis B virus, *HCC* hepatocellular carcinoma, *5ASA* 5-aminosalicylic acid

Among the six HBsAg-positive patients with altered transaminases, two patients had possible hepatitis B reactivation. Both of them were receiving steroid without antiviral prophylaxis at the time of altered transaminases. The first patient was put on budesonide 9 mg daily for 4 weeks and azathioprine (2 mg/kg) for mildly active ileocolonic Crohn’s disease. He was noted to have persistently elevated ALT up to 146 with elevated serum HBV DNA 7.85 log IU/mL. Steroid was stopped, azathioprine was changed to 6-mercaptopurine and mesalazine was added. Entecavir was started in view of possible hepatitis B reactivation and his serum HBV DNA level was subsequently undetectable and liver function remained normal. His underlying Crohn’s disease is well controlled with mesalazine and 6-mercaptopurine (0.5 mg/kg). Another patient had systemic steroid in private sector (exact duration and dosage unknown) and developed altered transaminases with highest ALT up to 2520 with HBV DNA 7.07 log IU/mL but no fulminant liver failure was observed. He was treated as hepatitis reactivation and lamivudine was started. His liver function was subsequently normalized and maintained normal. His underlying ulcerative colitis was well controlled with 5-ASA.

### Liver stiffness and HBV DNA levels results

We compared liver stiffness using Fibroscan and DNA levels of HBsAg-positive IBD patients were compared with HBsAg-positive controls without IBD. Fibroscan was performed in 21 of the 23 patients with both HBV and IBD. One patient was lost to follow up after initial inclusion and another patient refused Fibroscan. One patient (4.3 %) had confirmed liver cirrhosis by Fibroscan measurement. None had significant fibrosis. The mean liver stiffness in the cases was 5.8kPa (SD 2.3) while that in the matched controls was 7.0kPa (SD 3.3) (*p* = 0.081). The mean HBV DNA level was 3.76log IU/mL (SD 2.12) and 4.12log IU/mL (SD 2.15) (*p* = 0.319).in HBsAg-positive patients with IBD and without IBD, respectively.

## Discussion

Earlier studies have shown that patients with IBD have a higher prevalence of HBV than that of the general population [7]. In a recent paper fromChina, it was reported that the prevalence of past HBV infection in IBD patients was higher than that of non-IBD patients [21]. These may be attributed by the increased needof surgery and transfusion among IBD patients [21]. However, in a separatepaper it was shown that the prevalence rate of HBV infection in IBD patients was similar to that of the general population of China [22].

In Hong Kong, the prevalence of hepatitis B among IBD subjects was 5.7 % and this figure is comparable to the background population (around 7 %) [6].

Huang et al. reported that the prevalence of occult HBV infection in IBD patients was higher than that of non-IBD patients in Shanghai, China [21]. However, we included HBsAg-positive IBD patients but excluded IBD patients with antibodies to hepatitis B core antigen (anti-HBc) alone or occult HBV infection. Routine checking of anti-HBc to identify patients with occult HBV has been recommended for patients receiving chemotherapy [23]. However, acute hepatitis flare in occult HBV patients is very rare in patients receiving common medications for IBD unless patients were also receiving biologic agents [15]. Therefore, we sought to include only patients at a high risk of HBV reactivation.

Altered transaminases was not uncommon and was observed in 14.4 % of our IBD patients. Among those who were positive for HBV, 26 % had altered transaminases. The prevalence of altered transaminases among IBD patients with HBV was reported to be 36 % [15], 17 % [17] and 16 % [24], in studies from Spain, Korea and Hong Kong, respectively. The rate of altered transaminases among IBD patients with HBV is lower in Asia. The severity of altered transaminses appeared to be more severe in the Spanish population as 24 % of those with altered transaminases developed liver failure and half required liver transplantation [15], while <1 % was observed in a Korean cohort [17] but none in our cohort or in separate Chinese cohort [24].

Ng et al. reported that Caucasians are much more likely to receive steroid in the first year of diagnosis than that of Asian patients [2]. In addition, though not statistically significant, Caucasians with HBV also appeared to have a higher baseline HBV DNA than Asian patients [25]. The combination of these factors may explain the higher rate and severity of altered transaminases among Caucasian IBD patients.

Most international guidelines recommend that all HBV patients receiving immunosuppressants, regardless of the drug, dosage or duration, should receive anti-viral prophylaxis [11–14]. However, compliance rate with such strategy is low in our cohort. Among patients with positive HBsAg who received immunosuppressants, 38 % received anti-viral prophylaxis. In Hong Kong, there is limited or no reimbursement for antiviral prophylaxis and this likely explains the low compliance rate.

It is well established that steroids increase viral replication and is associated with HBV reactivation [26]. Based on our current findings, the risk of HBV reactivation appeared to be low in patients receiving immunosuppressive therapy in the form of thiopurines unless there was concomitant use of steroids. This finding echoes the recent update AGA guideline which recommends that only patients at moderate to high risk undergoing immunosuppressive therapy should have anti-viral prophylaxis [16]. According to the AGA guideline, chronic hepatitis B or occult hepatitis B patients treated with traditional immunosuppressive agents (e.g., azathioprine); chronic hepatitis B or occult hepatitis B treated with any dose of oral corticosteroids daily for ≤1 week were considered low risk for hepatitis B reactivation. In these patients, antiviral prophylaxis is not recommended by the AGA [15]. Based on our results and the latest AGA recommendation, we suggest that close monitoring of liver function and prompt initiation of anti-viral in case of altered transaminases may be a reasonable option for IBD patients with HBV treated with thiopurines.

We found that a past history of surgery and the use of steroids were associated with altered transaminases. The use of corticosteroid had been established to be risk factor for secondary steatohepatitis [27]. The use of total parental nutrition (TPN) may induce hepatosteatosis and result in altered transaminases. Although we did not have complete data on the use of TPN in our cohort, it is likely that patients with IBD receiving surgery would have received nutritional support during the pre and peri-operative period and this could account for the association of altered transaminases with surgery.

International guidelines recommend that all IBD patients should be screened for HBV [3, 28]. From our cohort, HBV infection per se was not associated with altered transaminases. Among the HBV patients with altered transaminases who were on immunosuppressant, only one patient on steroid and another patient on steroid and thiopurine had possible HBV reactivation. In the other cases with altered transaminases, there was no strong evidence of HBV reactivation.

Although more patients with HBV infection appeared to have used biologics this observation should be interpreted with caution in view of the small sample size. The frequency of use of other IBD medications including steroids, 5-ASA and immunosuppressant in Hong Kong was similar between HBV and non-HBV patients. In Korea, HBsAg-positive patients had a lower rate of use of immunosuppressant and had a worse outcome than HBsAg-negative IBD patients. The authors contributed the worse clinical outcome to the under-utilization of immunosuppressants in patients with both diseases because of the fear of HBV reactivation [18]. The lack of influence of HBV infection on the use of immunosuppressants may explain the similar clinical outcome related to IBD patients with HBV as compared to those without HBV. We therefore believe that physicians should prescribe IBD related medications to patients based on their disease control regardless of their HBV status so as to maintain good IBD control.

We did not exclude patients with primary sclerosing from our study. Our study investigated the cause of altered transaminases, as PSC predominantly affect alkaline phosphatase (ALP), instead of transaminases (ALT), including PSC in the cohort is unlikely to affect the results. For instance, the only patient with PSC in our cohort had cholestatic liver dysfucntion with normal ALT, hence it is unlikely to be a confounding factor of the study.

This study has several limitations. First, some of the patients have been started on anti-viral agents before the commencement of the study. Therefore the true effect of immunosuppressant in HBV patients may be masked. Prospective monitoring of the HBV DNA level and follow up of liver stiffness measurement in anti-viral naïve patients will provide further information on the true effect of immunosuppressant on HBV. Second, in Hong Kong, all hospital records have been computerized since early 2000, however, records before early 2000 may be incomplete and hence some cases of altered transaminases could have been missed because of unavailable data. Third, our cohort consisted of a modest number of patients with both IBD and HBV who were treated with immunosuppressants. Lastly, the size of the HBV cohort was modest and thus the analysis may be underpowered. However, the preliminary findings may shed light on the characteristics of this special group of IBD population.

## Conclusions

In conclusion, the prevalence of HBV among patients with IBD in Hong Kong was comparable to that of the general population. The use of steroids and a history of surgery were associated with altered transaminases in IBD patients. There was however no difference in the disease control and the choice or use of medications for the underlying disease between IBD subjects with or without HBV infection.

## Abbreviation

5ASA: 5-aminosaliylic acid
AGA: American Gastroenterological Association Institute
ALT: Alanine aminotransferase
CD: Crohn’s disease
CI: Confidence interval
CRP: C reactive protein
ESR: Erythrocyte sedimentation ratio
HBeAg: Hepatitis e antigen
HBsAg: Hepatitis B surface antigen
HBV: Hepatitis B virus
HCC: Hepatocellular carcinoma
IBD: Inflammatory bowel disease
OR: Odds ratio
SD: Standard deviation
TPN: Total parental nutrition
UC: Ulcerative colitis
ULN: Upper limit of normal
